# Soldering Hint

**Published:** 1902-07-15

**Authors:** L. C. Taylor

**Affiliations:** Hartford, Conn.


### SOLDERING HINT.

BY L. C. TAYLOR, D.D.S., HARTFORD, CONN.

When soldering crowns or bridges requiring a number of pieces of solder, first pickle well in acid so surfaces will be bright, then place solder in quantity about as needed, drop a few drops of sticky wax on it to hold solder in place, put borax over the wax, and heat up slowly to dry out. Then with blow-pipe bring the mass to a white heat, allowing the wax to burn out, and see how quickly the work will be completed without the solder crawling with borax or dropping from some slight jar of the case. The solder for a full plate can be put in place when plate is cold under the above method with great satisfaction. It has been suggested that the borax would flow with the wax, soak into investment, and crack the porcelain, but I have soldered with this method for more than a year and have never had a porcelain crack. Several friends to whom I have suggested this plan report that they are employing it all the time with gratifying results.—*Digest*.

